# New alkaloids from the fruiting bodies of *Ganoderma sinense*

**DOI:** 10.1007/s13659-011-0026-4

**Published:** 2011-11-23

**Authors:** Jie-Qing Liu, Cui-Fang Wang, Xing-Rong Peng, Ming-Hua Qiu

**Affiliations:** 1 State Key Laboratory of Phytochemistry and Plant Resources in West China, Kunming Institute of Botany, Chinese Academy of Sciences, Kunming, 650201 China; 2 College of Plant Protection, Yunnan Agricultural University, Kunming, 650224 China

**Keywords:** *Ganoderma sinense*, Ganodermataceae, alkaloids, sinensines

## Abstract

Four new alkaloids, sinensines B–E (**1**–**4**), together with one known alkaloid, sinensine (**5**), were isolated from the fruiting bodies of *Ganoderma sinense*. Their structures were elucidated on the basis of 1D and 2D NMR spectra analysis. The structure of sinensine E was confirmed by X-ray crystallographic analysis of its acetyl product (**4a**).

**Electronic Supplementary Material:**

Supplementary material is available for this article at 10.1007/s13659-011-0026-4 and is accessible for authorized users.

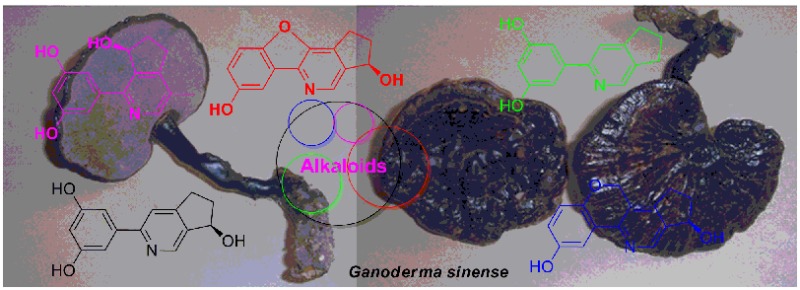

## Introduction

The fruiting bodies of *Ganoderma spp.*, commonly known as the Reishi mushroom, were widely used in China, Japan, and Korea as valuable crude drug, especially in the treatment of chronic hepatitis, nephritis, hepatopathy, neurasthenia, arthritis, bronchitis, asthma, gastric ulcer, and insomnia.[1] The chemical constituents of *Ganoderma spp.* comprised polysaccharides, fatty acids, alkaloids, nucleotides, proteins and peptides, trace elements, sterols, and triterpenoids.[2–4] Among them, triterpenoids were mainly responsible for the biological activities. It was reported that triterpenoids played an important role in antitumor,[5] inhibiting HIV-1 protease,[6] antiplasmodial,[7] antiviral,[8] and antimicrobial.[9]

Alkaloids were another chemical constituents obtained from *Ganoderma spp.* In 1990, ganoderma alkaloids A and B were isolated from the fruiting bodies of *G. capense* for the first time.[10] Researchers had detected ganoderma alkaloid by means of HPLC, but they did not find the trace of ganoderma alkaloid.[11] Until 2010, from the fruiting bodies of *G. sinense*, a novel alkaloid, named sinensine was isolated and exhibited activity in protecting the injury induced by hydrogen peroxide oxidation on HUVEC.[12]

To discover ganoderma alkaloids and evaluate their biological activities, amounts of the fruiting bodies of *G. sinense* conducted. On our progress of investigating the chemical constituents of *G. sinense*, three new triterpenoids with four-member ring displaying anti-tumor activity had been reported.[13] In this paper, the isolation and identification of four new alkaloids, sinensines B–E (**1**–**4**), together with one known alkaloid, sinensine (**5**) were reported. Their structures were identified mainly by 1D, 2D NMR spectra and X-ray analysis.

*To whom correspondence should be addressed. E-mail: mhchiu@mail.kib.ac.cn

## Results and Discussion

The fruiting bodies of *G. sinense* (50 kg) were extracted with 95% ethanol and filtered, concentrated in vaccum and partitioned with petroleum, ethyl acetate (EtOAc) and *n*-butanol, respectively. The EtOAc extract (1.5 kg) was repeatedly chromatographed on silica gel, RP-18, Sephadex LH-20, and HPLC to yield sinensines B–E (**1**–**4**) and sinensine (**5**).

The molecular formula of compound **1** was assigned as $C_{14}H_{13}NO_2$ ($[M + H]^+$; *m/z* 228.1003) by HRESIMS, which established nine degrees of unsaturation. The IR spectrum showed hydroxyl group (3383 $cm^{-1}$) and aromatic rings (1464 $cm^{-1}$). The $^1H$ NMR spectrum (Table 1) of **1** exhibited signals at $\delta_H$ 7.89 (d, 1H, $J$ = 2.4 Hz), 7.27 (2H, m), 8.21 (s, 1H), and 7.84 (s, 1H). The $^{13}C$ NMR spectrum (Table 1) showed fourteen carbon signals, including three methylene siganls at $\delta_C$ 32.9, 30.0 and 25.0; five $sp^2$ methine signals at $\delta_C$ 141.8, 119.6, 119.3, 115.9 and 113.5; six $sp^2$ quaternary-carbon signals at $\delta_C$ 156.2, 156.1, 153.6, 151.1, 138.5 and 120.4. These data of **1** were similar to those of sinensine (**5**)[12]. However, the 1D spectra of **1** displayed one more $sp^3$ methylene and $sp^2$ methine, as well as less a methyl and an oxymethine than those of **5**.

In $^1H$ NMR spectrum, the signals at $\delta_H$ 8.21 (s, 1H) and 7.84 (s, 1H) belonging to pyridine indicated that two olefinic carbons were located at C-3 and C-7. So, the location of cyclopentenyl group in **1** was different with that of in **5**. The HMBC correlations from H-7 ($\delta_H$ 8.21) to C-2 ($\delta_C$ 156.2) and C-9 ($\delta_C$ 138.5), from H-3 ($\delta_H$ 7.84) to C-1' ($\delta_C$ 120.4), C-9 ($\delta_C$ 138.5) and C-8 ($\delta_C$ 156.1), from H-4 ($\delta_H$ 2.67) to C-9 ($\delta_C$ 138.5), C-8 ($\delta_C$ 156.1) and C-3 ($\delta_C$ 115.9), from H-6 ($\delta_H$ 2.64) to C-7 ($\delta_C$ 141.8), C-9 ($\delta_C$ 138.5) and C-8 ($\delta_C$ 156.1), and from H-5 ($\delta_H$ 1.78) to C-9 ($\delta_C$ 138.5), C-8 ($\delta_C$ 156.1), C-4 ($\delta_C$ 32.9), and C-6 ($\delta_C$ 30.0) confirmed that the cyclopentenyl group was cyclized with pyridine at C-8 and C-9.

sinensine B (**1**): R=H
sinensine C (**2**): R=OH

sinensine D (**3**)

sinensine E (**4**) R = H
diacetyl sinensine E (**4a**) R = Ac sinensine (**5**)

A dihydroxyl phenyl group at C-2 was deduced by the HMBC correlations from H-2' ($\delta_H$ 7.27) to C-1' ($\delta_C$ 120.4) and C-3' ($\delta_C$ 153.6), and from H-3 ($\delta_H$ 7.84) to C-1', C-9 and C-8. The locations of two hydroxyl group at C-3' and C-5' were assigned by the $^1$H NMR spectrum and the coupling constants of protons. H-2', H-4' and H-6' with coupling constants of no more than 3.0 Hz indicated that they were at *meta*-positions. Thus, sinensine B **(1)** was established.

Sinensine C **(2)** possessed a molecular formula $C_{14}H_{13}NO_3$ by HRESIMS ($[M + H]^+$; *m/z* 244.0966, calcd 244.0973), which differed from **1** for one more oxygen atom. By detail comparison of 1D NMR data of **2** and **1**, they were similar, except for the absence of a methylene and the presence of an oxymethine. Above data indicated that an additional hydroxyl group in **2** was attached to cyclopentenyl group. The further proof was established from the HMBC correlations from H-4 ($\delta_H$ 2.93 and 2.66) to C-9 ($\delta_C$ 141.5), C-8 ($\delta_C$ 157.2) and C-3 ($\delta_C$ 116.3), from H-5 ($\delta_H$ 2.42 and 2.13) to C-9, C-8, C-4 ($\delta_C$ 30.4), and C-6 ($\delta_C$ 73.7), and from H-6 ($\delta_H$ 5.48) to C-7 ($\delta_C$ 142.8), C-9 and C-8. This illustrated that the hydroxyl group was located at C-6. Thus, the plantar structure of compound **2** was identified.

Molecular formula $C_{14}H_{11}NO_3$ of compound **3** was determined by HRESIMS. Its IR spectrum showed presence of hydroxyl group (3445 cm$^{-1}$) and aromatic rings (1614 and 1482 cm$^{-1}$). On comparison of the 1D NMR data between **3** and **2**, the signals due to the phenyl group and the group of cyclopenta[b]pyridine could be observed. However, the 1D NMR spectra of **3** showed a down-field *sp*$^2$ quaternary carbon signal ($\delta_C$ 160.8) instead of an up-field *sp*$^2$ methine signal in **2**, which suggested that an *sp*$^2$ methine in **2** was oxidized to an *sp*$^2$ quaternary carbon in **3**.

In the $^1$H-$^1$H COSY spectrum, cross peak between signals at 8.56 (d, $J$ = 2.0 Hz) and 7.35 (2H, overlapped) indicated that these signals belonged to the phenyl group. This deduction would be confirmed by the HMBC correlations from H-3' ($\delta_H$ 7.35), H-4' ($\delta_H$ 7.35) and H-6' ($\delta_H$ 8.56) to C-2' ($\delta_C$ 155.8), C-5' ($\delta_C$ 146.5), and C-1' ($\delta_C$ 123.1). Meanwhile, analysis of their coupling constants suggested that the phenyl group was an ABC system, which demonstrated that **3** had a 1',2',5'-trisubstituted phenyl group.

Since there were two oxidized olefinic carbons at the phenyl group, the additional oxidized olefinic carbon could be located at the pyridine. Meanwhile, in the HMBC spectrum, the correlations of H-7 with C-8, C-9 and C-6 indicated that the additional oxidized olefinic carbon was at C-3. Base on the molecular formula $C_{14}H_{11}NO_3$ (10 degree of unsaturation) of **3**, we deduced that C-2' and C-3 formed an ether bond. Furthermore, *ChemDraw 3D* model of **3** (Figure 1) showed that the phenyl group, the furan and the pyridine were formed a conjugate

**Table 1. $^1$H (500 MHz, $C_5D_5N$, $J$ in Hz) and $^{13}$C NMR (125 MHz, $C_5D_5N$) data for compounds 1–4.**

| No. | sinensine B (**1**) | | sinensine C (**2**) | | sinensine D (**3**) | | sinensine E (**4**) | |
|---|---|---|---|---|---|---|---|---|
| | $\delta_H$ | $\delta_C$, type | $\delta_H$ | $\delta_C$, type | $\delta_H$ | $\delta_C$, type | $\delta_H$ | $\delta_C$, type |
| 2 | | 156.2, C | | 155.4, C | | 115.1, C | | 154.1, C |
| 3 | 7.84, s | 115.9, CH | 7.90, s | 116.3, CH | | 160.8, C | | 123.5, C |
| 4a | 2.67, overlapped | 32.9, $CH_2$ | 2.93, ddd | 30.4, $CH_2$ | 3.74, ddd | 31.9, $CH_2$ | 2.80, ddd | 27.5, $CH_2$ |
| | | | (16.7, 8.5, 4.4) | | (18.7, 8.8, 4.3) | | (16.1, 8.5, 4.4) | |
| 4b | | | 2.66, m | | 3.35, td (18.7, 7.8) | | 2.53, m | |
| 5a | 1.78, m | 25.0, $CH_2$ | 2.42, m | 36.3, $CH_2$ | 2.53, m | 36.1, $CH_2$ | 2.43, m | 36.3, $CH_2$ |
| 5b | | | 2.13, m | | 2.24, m | | 2.15, m | |
| 6 | 2.64, overlapped | 30.0, $CH_2$ | 5.48, br. s | 73.7, CH | 5.57, t (6.4) | 73.7, CH | 5.51, t (6.0) | 73.9, CH |
| 7 | 8.21, s | 141.8, CH | 8.68, s | 142.8, CH | 9.19, s | 151.8, CH | 8.91, s | 145.8, CH |
| 8 | | 156.1, C | | 157.2, C | | 157.4, C | | 148.4, C |
| 9 | | 138.5, C | | 141.5, C | | 144.4, C | | 142.7, C |
| 10 | | | | | | | 5.20, d (2.6) | 65.7, $CH_2$ |
| 1' | | 120.4, C | | 120.4, C | | 123.1, C | | 125.1, C |
| 2' | 7.27, overlapped | 119.6, CH | 7.27, overlapped | 119.9, CH | | 155.8, C | | 150.0, C |
| 3' | | 153.6, C | | 153.6, C | 7.35, overlapped | 120.7, CH | 7.12, d (8.6) | 118.9, CH |
| 4' | 7.89, d (2.4) | 113.5, CH | 7.89, s | 113.6, CH | 7.35, overlapped | 118.2, CH | 7.22, m | 118.3, CH |
| 5' | | 151.1, C | | 151.2, C | | 146.5, C | | 147.8, C |
| 6' | 7.27, overlapped | 119.3, CH | 7.27, overlapped | 119.4, CH | 8.56, d (2.0) | 110.5, CH | 8.44, d (2.6) | 111.8, CH |

surface. The conjugate surface could result in the carbon signal of C-2 upfield shift to 115.1 *ppm*. However, *ChemDraw 3D* figures of **2** and **4** did not display the conjugate surface, so the chemical shifts of C-2 in **2** and **4** were about 155 *ppm*. Finally, the structure of **3** was deduced.

**Figure 1.** The *ChemDraw 3D* figures of **2**–**4**.

The molecular formula of **4** was established as $C_{15}H_{13}NO_3$ by the HRESIMS. The $^{1}$H and $^{13}$C NMR spectra of **4** revealed the siganls at $\delta_H$ 8.44 (d, 1H, $J$ = 2.6 Hz), 7.12 (d, 1H, $J$ = 8.6 Hz) and 7.22 (m, 1H), and siganls at $\delta_C$ 150.0, 147.8, 125.1, 118.9, 118.3 and 111.8 due to the 1',2',5'-trisubstituted phenyl group. Carefully compared the 1D NMR between **3** and **4**, an oxymethylene signal ($\delta_H$ 5.20 and $\delta_C$ 65.7) in **4** was observed and a downfield carbon signal at 160.8 *ppm* in **3** was instead of a upfield carbon signal in **4**, which suggested that the oxymethylene of **4** was instead of the hydroxyl group of **3** at C-3. Above deduction and the presence of an ether bond between C-2' and C-10 were confirmed by the HMBC correlations from H-10 ($\delta$ 5.20) to C-3 ($\delta$ 123.5), C-2' ($\delta$ 150.0) and C-8 ($\delta$ 148.4).

In order to further verify the structure of **4** deduced above and to describe the configuration of **4**, the single-crystal X-ray would be helpful. However, **4** was not easy to crystallize in most kinds of organic solvent. Fortunately, acetylization of **4** afforded 5',6-diacetyl sinensine E (**4a**) crystallized in MeOH/$H_2O$ solvent. Consequently, a single X-ray crystallographic diffraction[14] of **4a** was conducted as shown in Figure 2. The relative configuration of OH-6 was deduced as $\beta$-oriented. Based on above evidence, the structure of **4** was assigned.

On the basis of the specific rotations of **2**, **3** and **4**, it was illustrated that the relative configurations of OH-6 in **2** and **3** were determined to be $\beta$, the same as that of **4**.

## Experimental Section

**General Experimental Procedures**. Petroleum ether (PE) for chromatography had a b.p. range of 60–90°. Column chromatography (CC): silica gel ($SiO_2$, 200–300 mesh; Qingdao Marine Chemical, Inc.); Lichroprep RP-18 (40–63 $\mu$m; Merck), or Sephadex LH-20 (Pharmacia). Optical rotations: Jasco P-1020 spectropolarimeter. IR spectra: Bruker Tensor 27 instrument, KBr pellets. NMR Spectra: Bruker AV-400 and DRX-500 instruments. HRESIMS: API Qstar Pulsar i spectrometer; in *m/z*.

**Figure 2.** Single-crystal X-ray structure of **4a**.

**Plant Material.** The fruiting bodies of *G. sinense* were purchased from Yunnan Nanhua wild mushroom market, and were identified by Professor Pei-Gui Liu, who was mushroom taxonomist in Kunming Institute of Botany, Chinese Academy of Sciences.

**Extraction and Isolation.** The dry fungus (50 kg) was ground and extracted in EtOH at 70 ℃ for three times. The extract was then decanted and evaporated, and the residue was extracted with EtOAc. The EtOAc extract was fractionated by CC (silica gel; $CHCl_3$/MeOH step gradients): Fractions 1–4 ($CHCl_3$/MeOH : 100:1, 50:1, 20:1, 5:1). Fraction 3 ($CHCl_3$/MeOH : 20:1) was subjected to reverse-phase silica gel (MeOH/$H_2O$ step gradients: 45% $\rightarrow$ 80%). 60% fraction was treated by CC (silica gel; Sepadex LH-20; reverse-phase HPLC and prep. TLC) and finally gave **1** (18 mg), **2** (19 mg), **3** (45 mg), **4** (35 mg) and **5** (15 mg).

**Sinensine B (1):** white powder ($CHCl_3$/MeOH); $[\alpha]_D^{25} - 8.18$ ($c$ 0.1, $CHCl_3$/MeOH); UV (MeOH/$CHCl_3$, $\lambda_{max}$ (log ε)): 266 (4.20), 345 (2.98) nm; IR (KBr) $\nu_{max}$ 3383, 2965, 2930, 2851, 1464 $cm^{-1}$; $^{1}$H and $^{13}$C NMR: see Table 1; ESIMS *m/z* 228 $[M + H]^+$; positive ion HRESIMS *m/z* 228.1003 $[M + H]^+$ (calcd. for $C_{14}H_{14}NO_2$, 228.1024).

**Sinensine C (2):** white powder ($CHCl_3$/MeOH); $[\alpha]_D^{25} - 11.08$ ($c$ 0.1, $CHCl_3$/MeOH); UV (MeOH/$CHCl_3$, $\lambda_{max}$ (log ε)): 268 (4.33), 333 (3.01) nm. IR (KBr) $\nu_{max}$ 3445, 2978, 1614, 1482, 1444 $cm^{-1}$; $^{1}$H and $^{13}$C NMR: see Table 1; ESIMS *m/z* 244 $[M + H]^+$; positive ion HRESIMS *m/z* 244.0966 $[M + H]^+$ (calcd. for $C_{14}H_{14}NO_3$, 244.0973).

**Sinensine D (3):** yellow powder ($CHCl_3$/MeOH); $[\alpha]_D^{25} - 11.08$ ($c$ 0.1, $CHCl_3$/ MeOH); UV (MeOH/$CHCl_3$, $\lambda_{max}$ (log ε)): 266 (4.28), 341 (3.12) nm; IR (KBr) $\nu_{max}$ 3445, 2978, 1614, 1482, 1444 $cm^{-1}$; $^{1}$H and $^{13}$C NMR: see Table 1; ESIMS *m/z* 264 $[M + Na]^+$; positive ion HRESIMS *m/z* 264.2314 $[M + Na]^+$ (calcd. for $C_{14}H_{11}NO_3Na$, 264.2318).

**Sinensine E (4):** yellow powder ($CHCl_3$/MeOH); $[\alpha]_D^{25} - 7.00$ ($c$ 0.1, $CHCl_3$/MeOH); UV (MeOH/$CHCl_3$, $\lambda_{max}$ (log ε)):

265 (4.17), 344 (3.20) nm. IR (KBr) $v_{max}$ 3416, 3267, 1611, 1575, 1470, 1428 $cm^{-1}$; $^{1}H$ and $^{13}C$ NMR: see Table 1; ESIMS *m/z* 256 $[M + H]^+$; positive ion HRESIMS *m/z* 256.0974 $[M + H]^+$ (calcd. for $C_{15}H_{14}NO_3$, 256.0973).

**Acetylization of 4 to 4a:**

**Crystal Data for 4a:** $C_{19}H_{17}NO_5$; $M$ = 339.34, colorless piece, space group $P2_12_12$, $a$ = 11.8937 (3) Å, $b$ = 28.7658 (6) Å， $c$ = 5.86480 (10) Å, $\alpha = \beta = \gamma = 90°$, $V$ = 2006.53 (7) $Å^3$, $Z$ = 4, $d$ = 1.446 $mg/cm^3$, crystal dimensions 0.68 × 0.63 × 0.14 mm was used for measurements on a Bruker APEX DUO diffractometer with a graphite monochromator (Φ/ω scans, $2\theta_{max}$ = 60.08°), Mo $K_\alpha$ radiation. The total number of independent reflections measured was 16187, of which 4180 were observed ($|F|^2 \geq 2\sigma|F|^2$). Final indices: $R_1$ = 0.0331, $wR_2$ = 0.0877 ($w = 1/\sigma|F|^2$)， $S$ = 1.074. The crystal structure of **4a** was solved by direct method SHELXS-97 (Sheldrich, G. M. University of Gottingen; Gottingen, Germany, 1997) and the full-maxtrix least-squares deposited in the Cambridge Crystallographic Data Centre (deposition number: 847702). Copies of these data can be obtained free of charge via the Internet at www.ccdc.cam.ac.uk/conts/retrieving.html (or from the Cambridge Crystallographic Data Center, 12 Union Road, Cambridge CB2 1EZ, U.K.; fax (+44) 1223-336-033; or e-mail: deposit @ccdc.cam.ac.uk).

**Electronic Supplementary Material**

Supplementary material is available in the online version of this article at http://dx.doi.org/10.1007/s13659-011-0026-4 and is accessible for authorized users.

**Acknowledgments**

The project was financially supported by the General Program of NSFC (No. 81172940) and Knowledge Innovation Program of the CAS (Grant No. KSCX2-YW-G-038, KSCX2-YW-R-194, KZCX2-XB2-15-03,), as well as Foundation of State Key Laboratory of Phytochemistry and Plant Resources in West China (P2010-ZZ14).

**Open Access** This article is distributed under the terms of the Creative Commons Attribution License which permits any use, distribution, and reproduction in any medium, provided the original author(s) and source are credited.

## Electronic supplementary material

Supplementary material, approximately 708 KB.
