# Supplementary material for: New alkaloids from the fruiting bodies of Ganoderma sinense
Source: Nat Prod Bioprospect. 2011 Nov 23;1(2):93–6. doi: 10.1007/s13659-011-0026-4 (PMC4131651; doi:10.1007/s13659-011-0026-4)

Electronic Supplementary Material

New alkaloids from the fruiting bodies of *Ganoderma sinense*

Jie-Qing LIU,[a] Cui-Fang WANG,[a] Xing-Rong PENG,[a,b] and Ming-Hua QIU[a,]*

[a]State Key Laboratory of Phytochemistry and Plant Resources in West China, Kunming Institute of Botany, Chinese Academy of Sciences, Kunming 650201, China

[b]College of Plant Protection, Yunnan Agricultural University, Kunming 650224, China

Received 16 October 2011; Accepted 14 November 2011

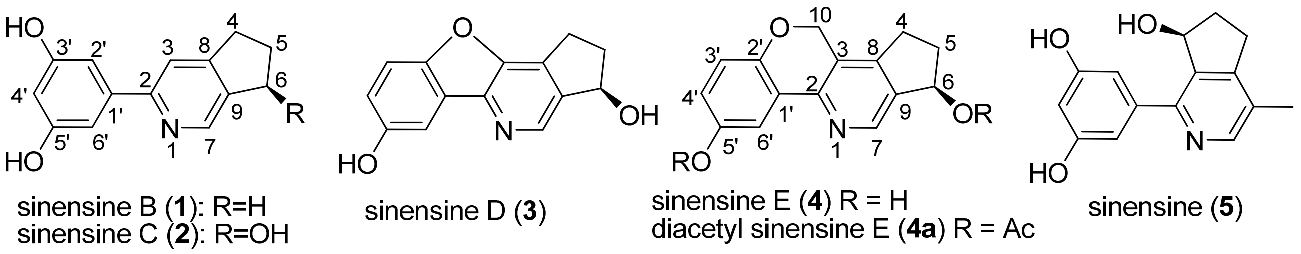

Structures of compounds **1**–**5**.

*To whom correspondence should be addressed. E-mail: mhchiu @mail.kib.ac.cn.

S1. ^{1}H NMR (500 MHz, C_5D_5N) spectra of the new compound sinensine B (**1**)……………….3
S2. ^{13}C NMR (125 MHz, C_5D_5N) spectra of the new compound sinensine B(**1**). ……………….4
S3. The HMBC spectra of the new compound sinensine B (**1**). ………………. ………. ………5
S4. The HSQC spectra of the new compound sinensine B (**1**). ………………. ………. ……….6
S5. ^{1}H-^{1}H COSY spectra of the new compound sinensine B (**1**). ………………. ………. ……7
S6. ^{1}H NMR (500 MHz, C_5D_5N) spectra of the new compound sinensine C (**2**). ……………….8
S7. ^{13}C NMR (125 MHz, C_5D_5N) spectra of the new compound sinensine C (**2**). ……………….9
S8. The HMBC spectra of the new compound sinensine C (**2**). ………………………. ………..10
S9. The HSQC spectra of the new compound sinensine C (**2**). ………………. ………. ……….11
S10. ^{1}H-^{1}H COSY spectra of the new compound sinensine C (**2**). ………………………. ………12
S11. ^{1}H NMR (500 MHz, C_5D_5N) spectra of the new compound sinensine D (**3**). ……………….13
S12. ^{13}C NMR (125 MHz, C_5D_5N) spectra of the new compound sinensine D (**3**). ……………….14
S13. The HMBC spectra of the new compound sinensine D (**3**). ……………………. ………..…15
S14. The HSQC spectra of the new compound sinensine D (**3**). ……………………. ………..…16
S15. ^{1}H-^{1}H COSY spectra of the new compound sinensine D (**3**). ……………………. ………..…17
S16. ^{1}H NMR (500 MHz, C_5D_5N) spectra of the new compound sinensine E (**4**). ……………….18
S17. ^{13}C NMR (125 MHz, C_5D_5N) spectra of the new compound sinensine E (**4**). ……………….19
S18. The HMBC spectra of the new compound sinensine E (**4**). …………………. ……….…….20
S19. The HSQC spectra of the new compound sinensine E (**4**). …………………. ……….…….21
S20. ^{1}H-^{1}H COSY spectra of the new compound sinensine E (**4**). ………………. ……….……….22

S1. ^{1}H NMR (500 MHz, C_5D_5N) spectra of the new compound sinensine B.

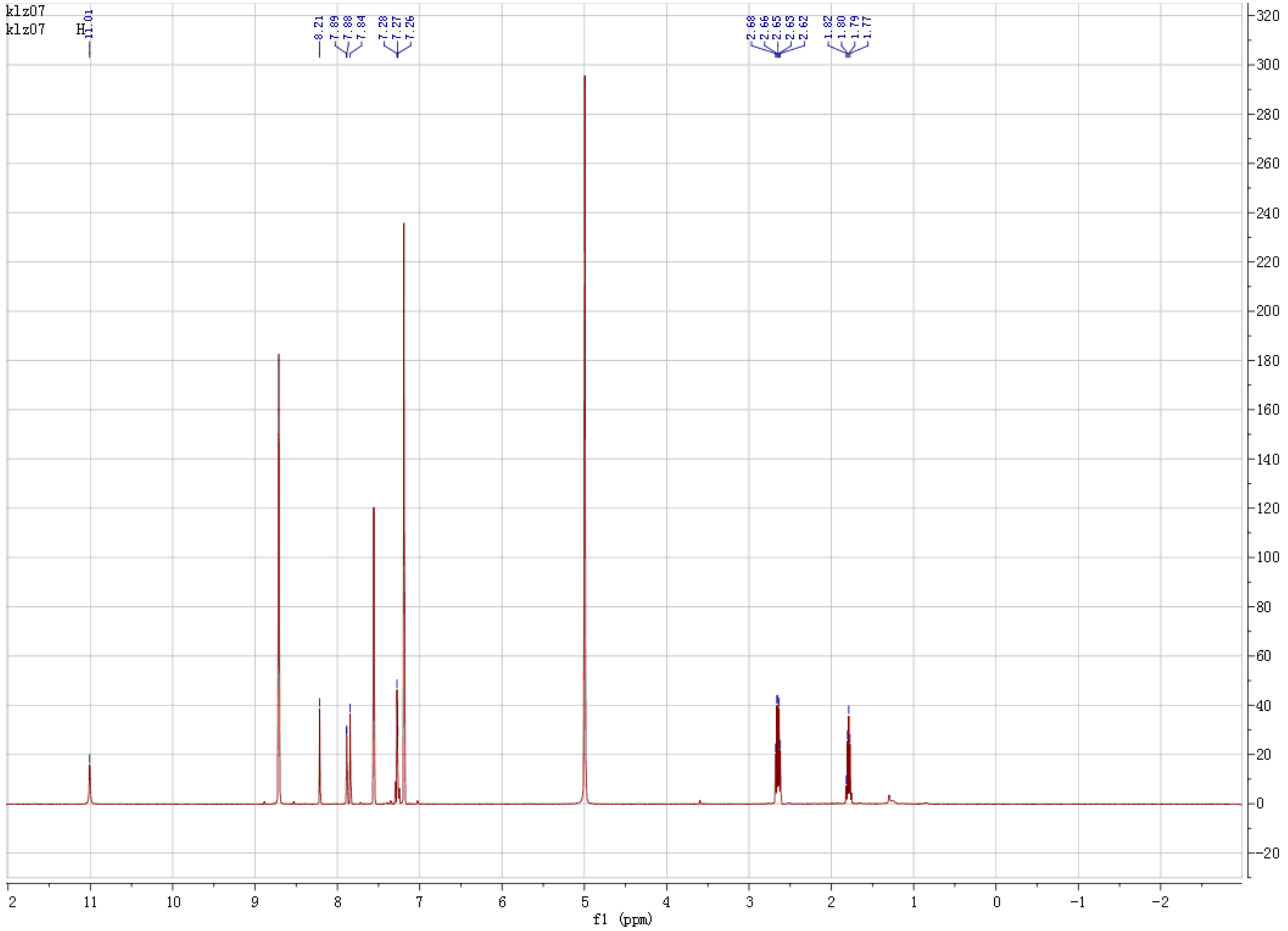

S2. ^{13}C NMR (125 MHz, C_5D_5N) spectra of the new compound sinensine B.

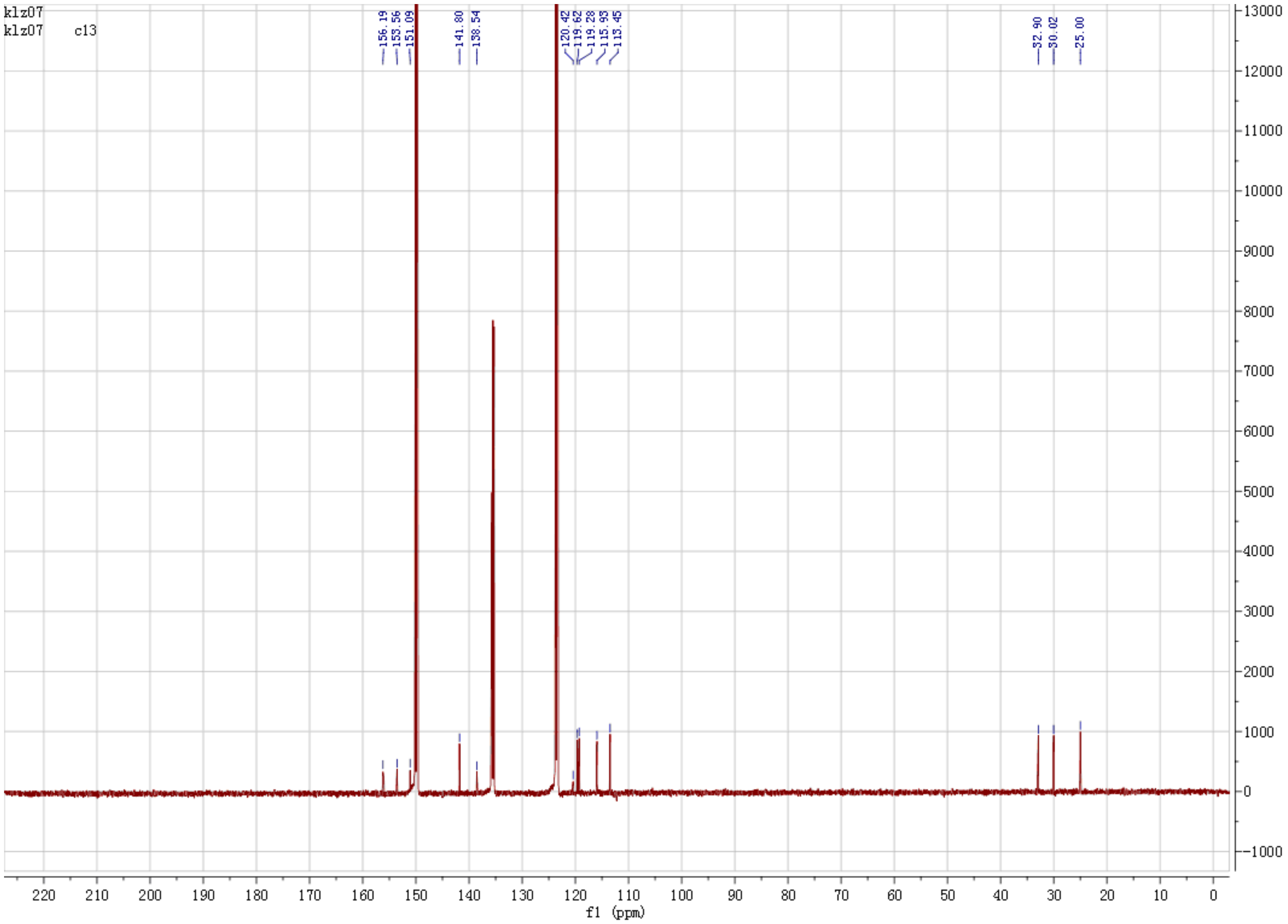

S3. The HMBC spectra of the new compound sinensine B.

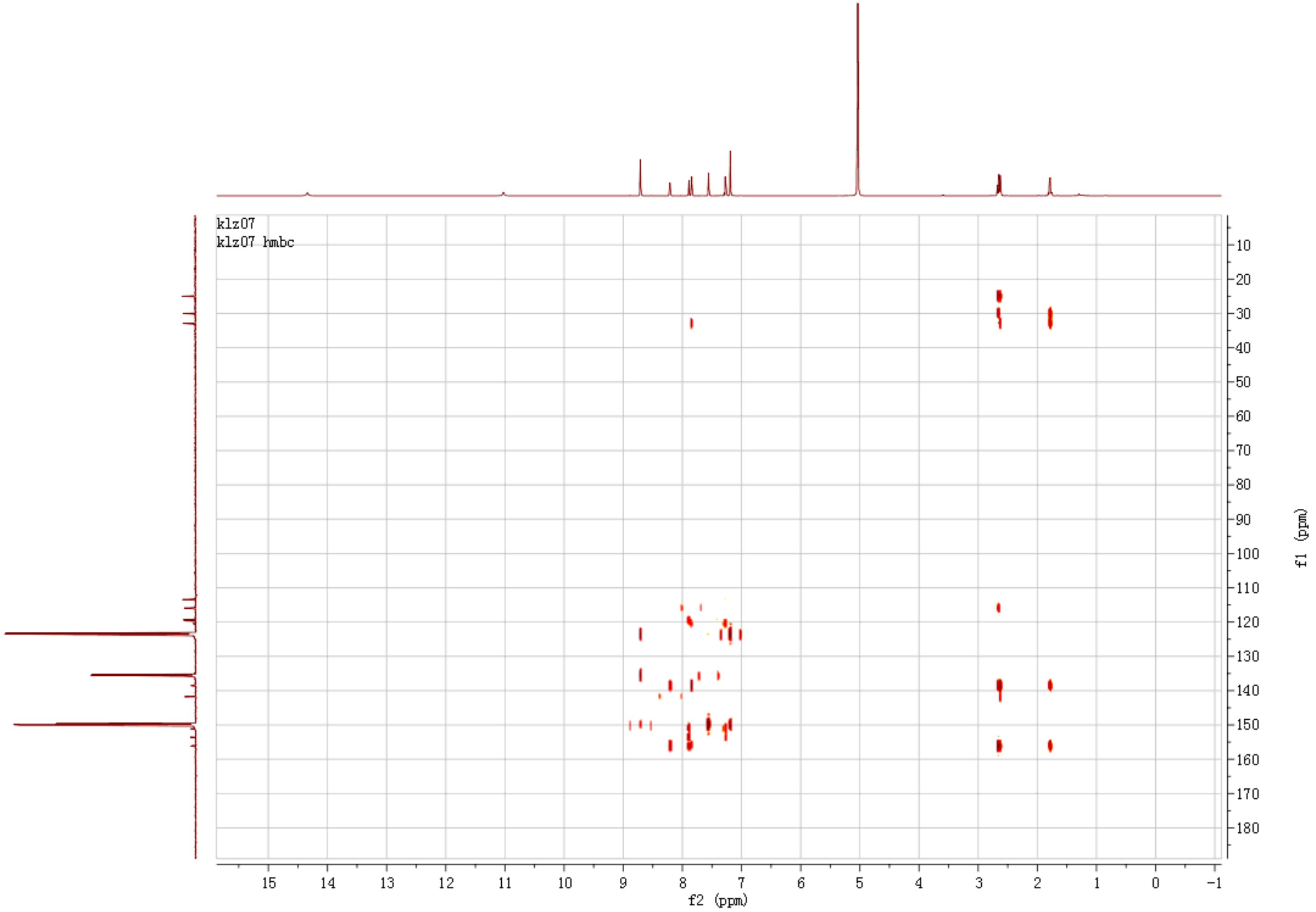

S4. The HSQC spectra of the new compound sinensine B.

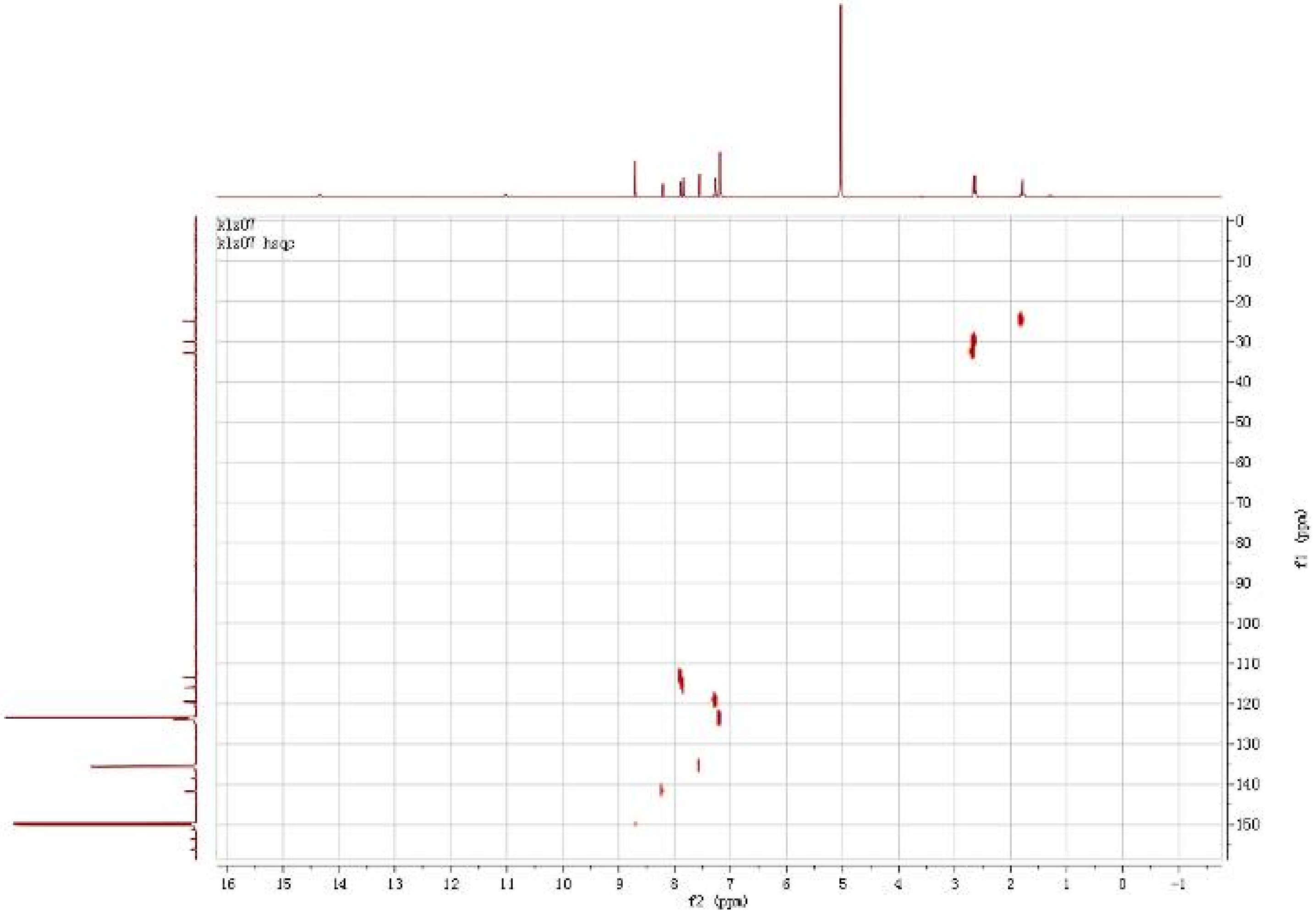

S5. ^{1}H-^{1}H COSY spectra of the new compound sinensine B.

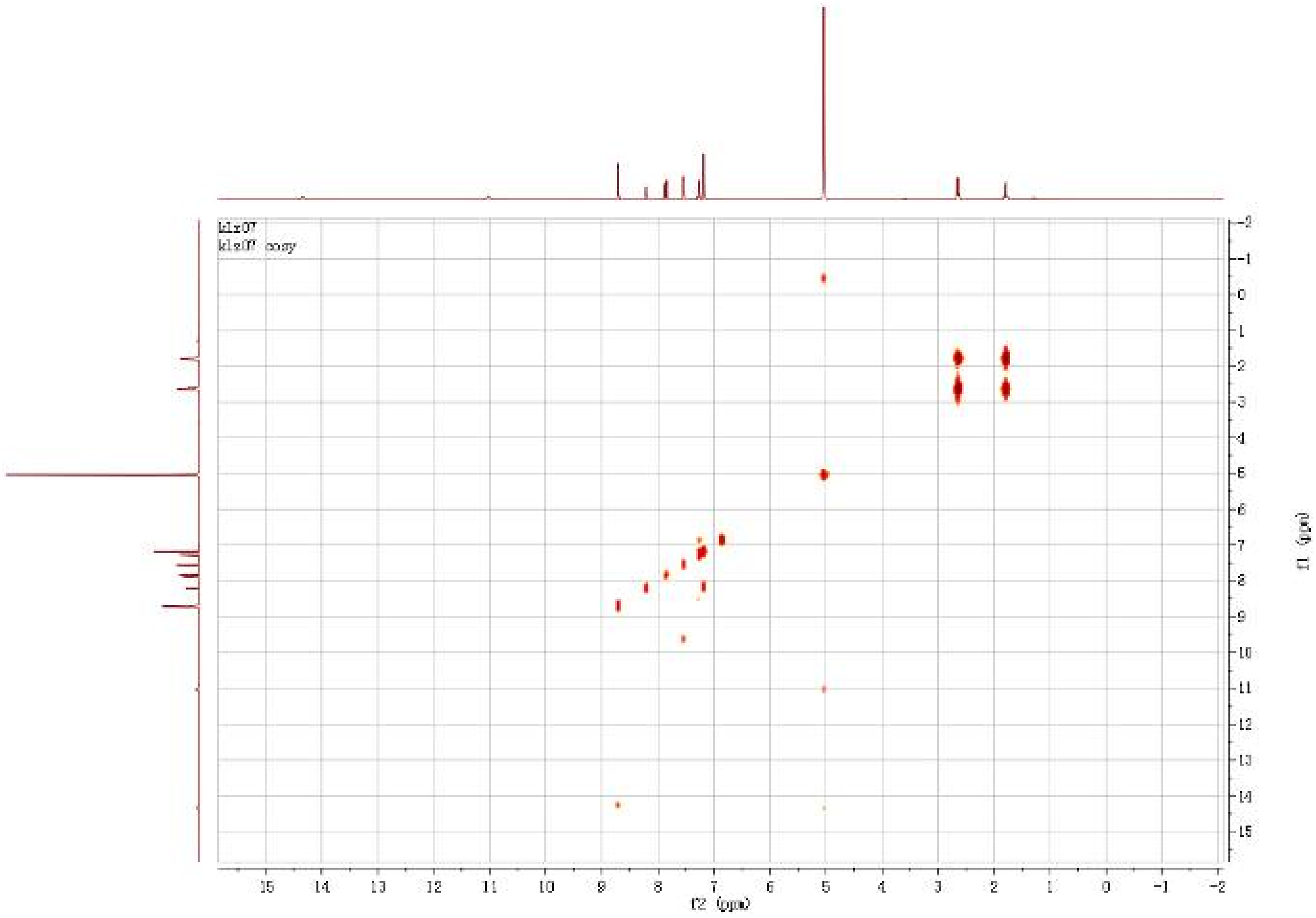

S6. ^{1}H NMR (500 MHz, C_5D_5N) spectra of the new compound sinensine C.

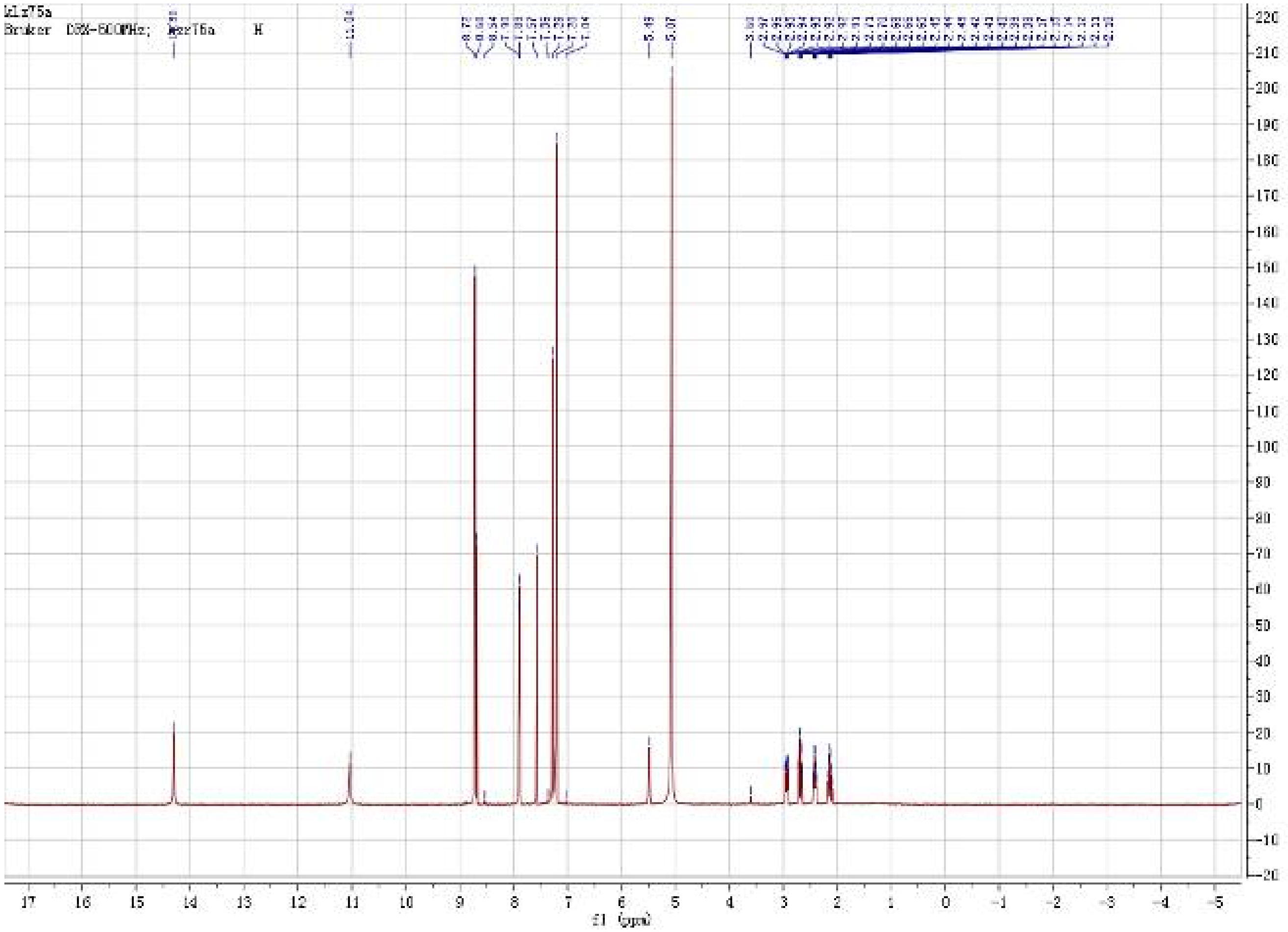

S7. ^{13}C NMR (125 MHz, C_5D_5N) spectra of the new compound sinensine C.

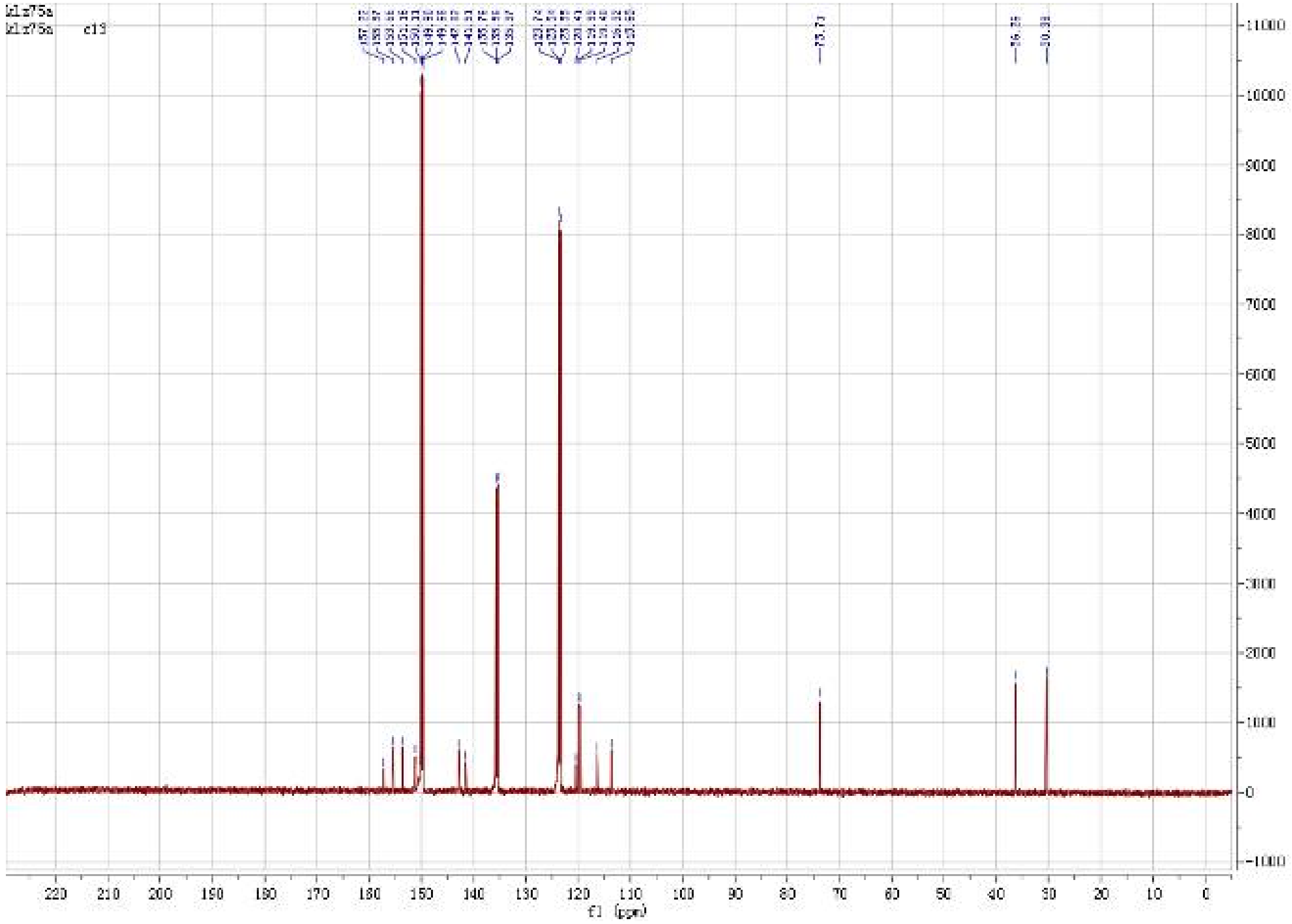

S8. The HMBC spectra of the new compound sinensine C.

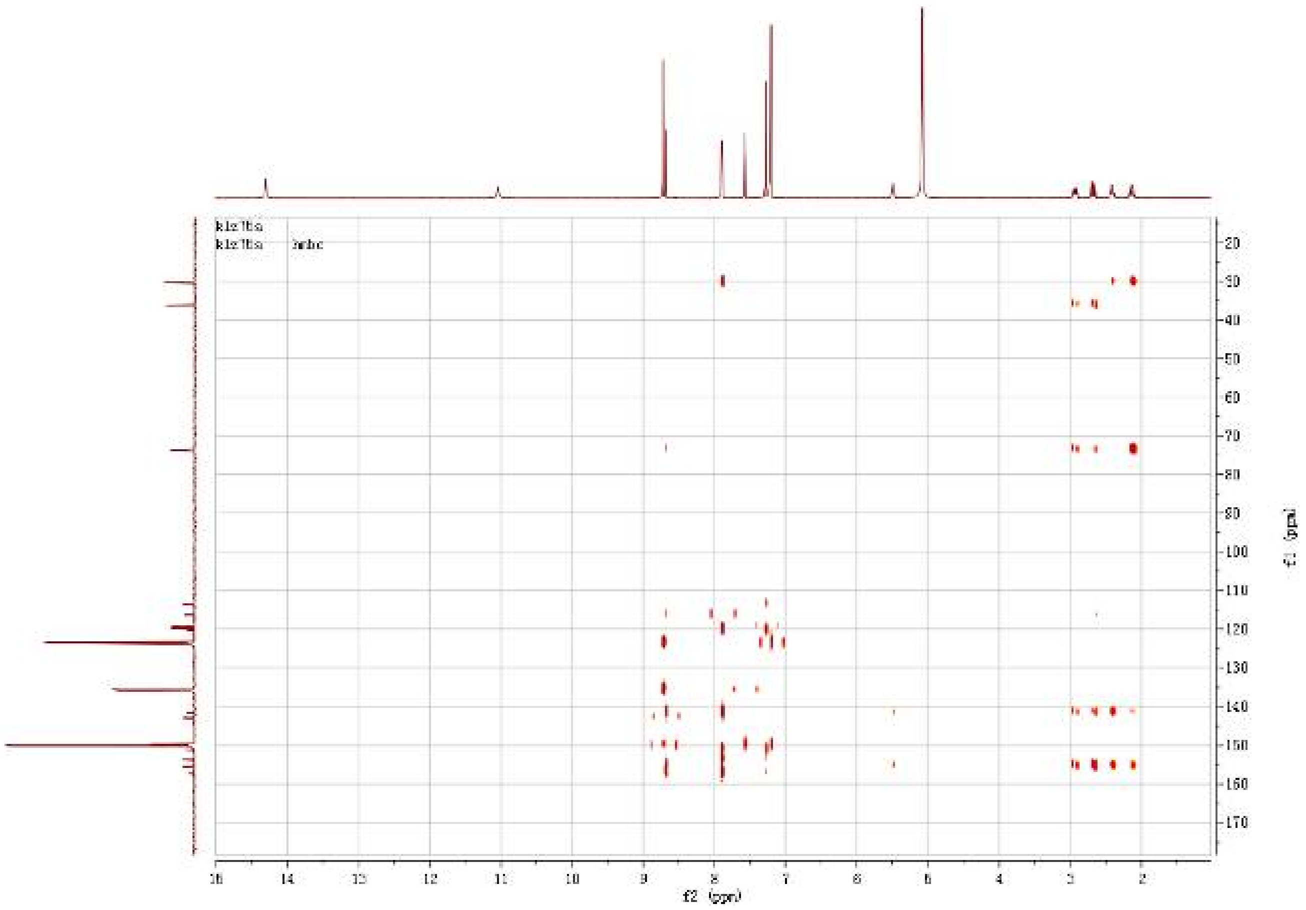

S9. The HSQC spectra of the new compound sinensine C.

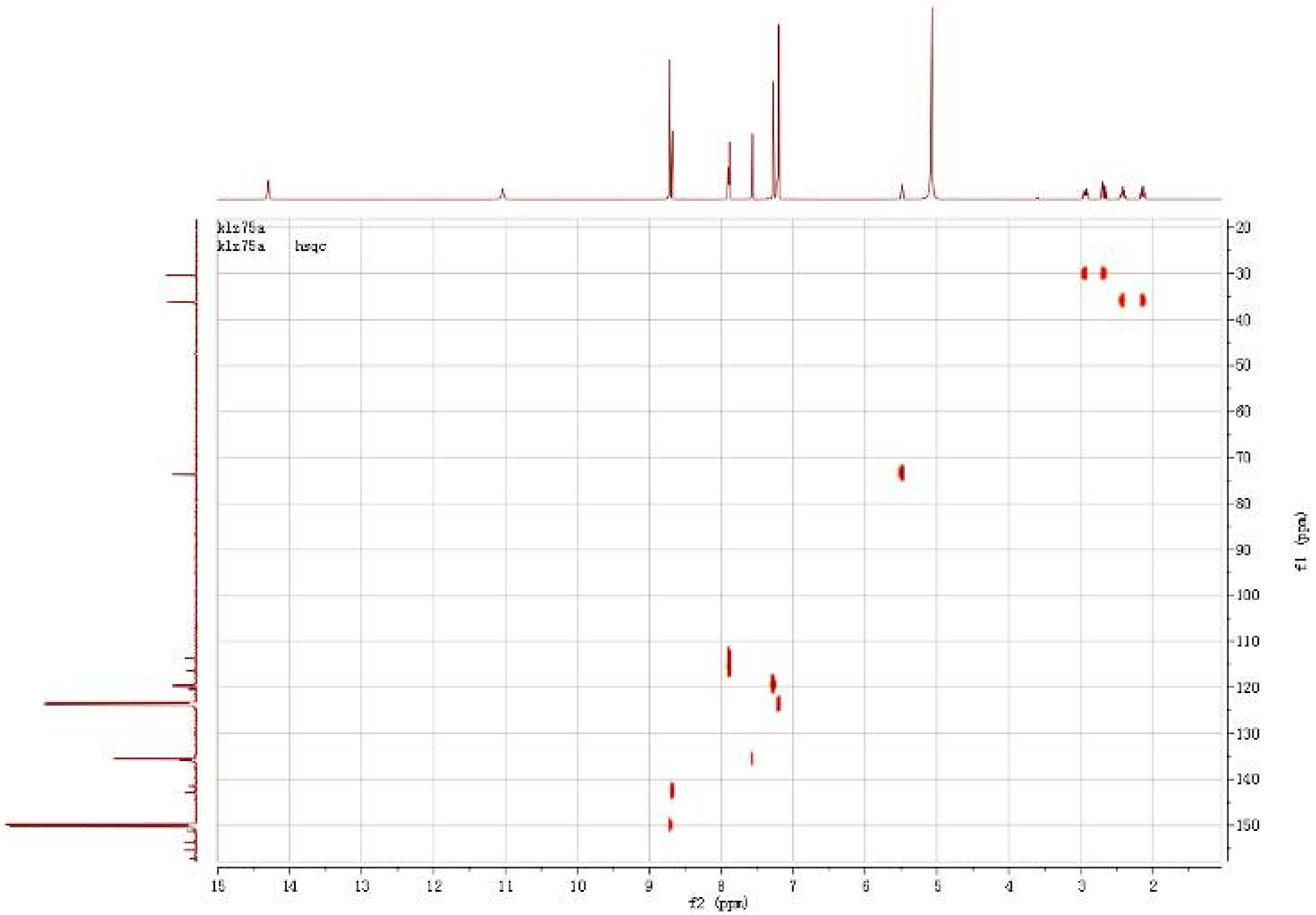

S10. ^{1}H-^{1}H COSY spectra of the new compound sinensine C.

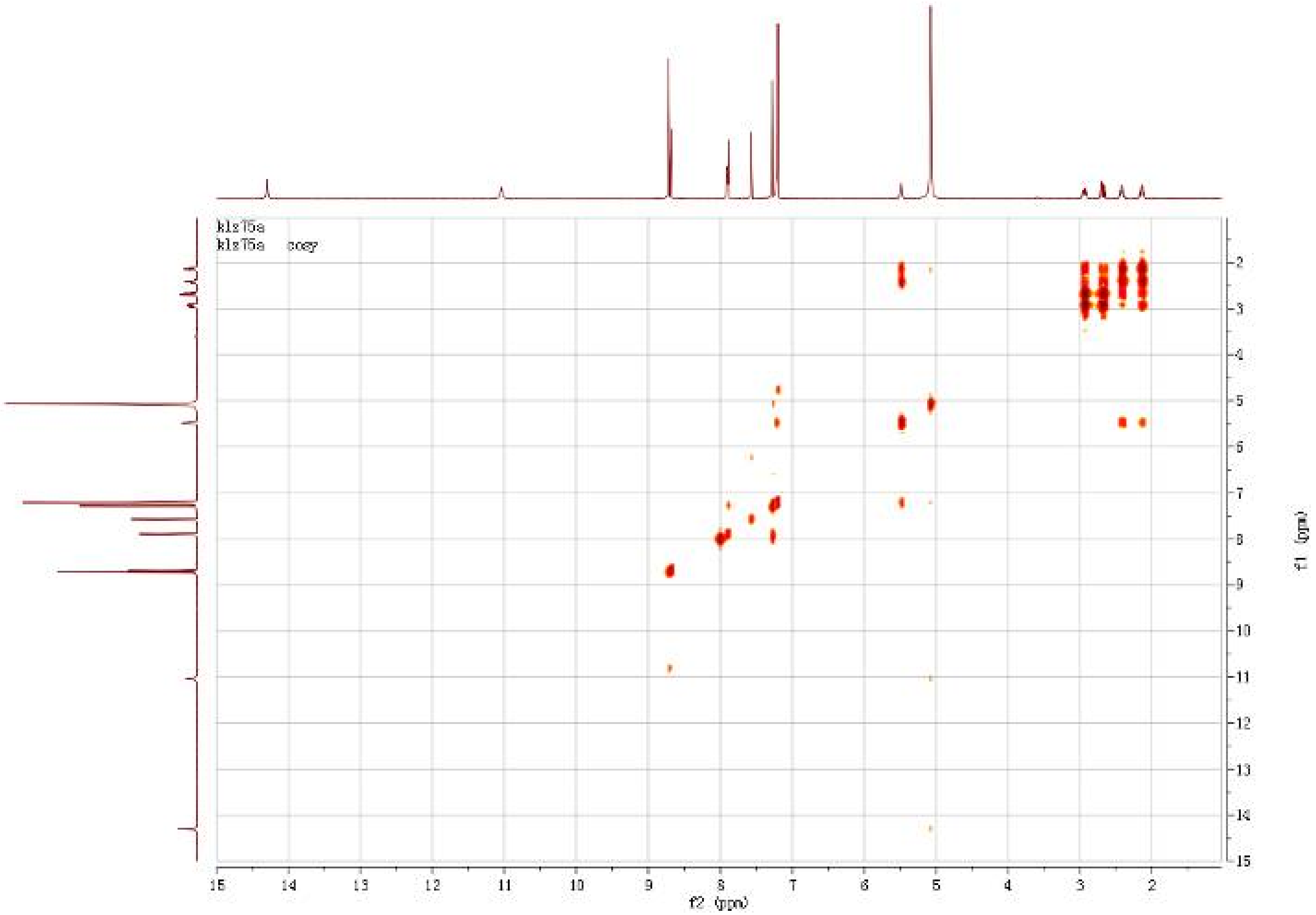

S11. ^{1}H NMR (500 MHz, C_5D_5N) spectra of the new compound sinensine D.

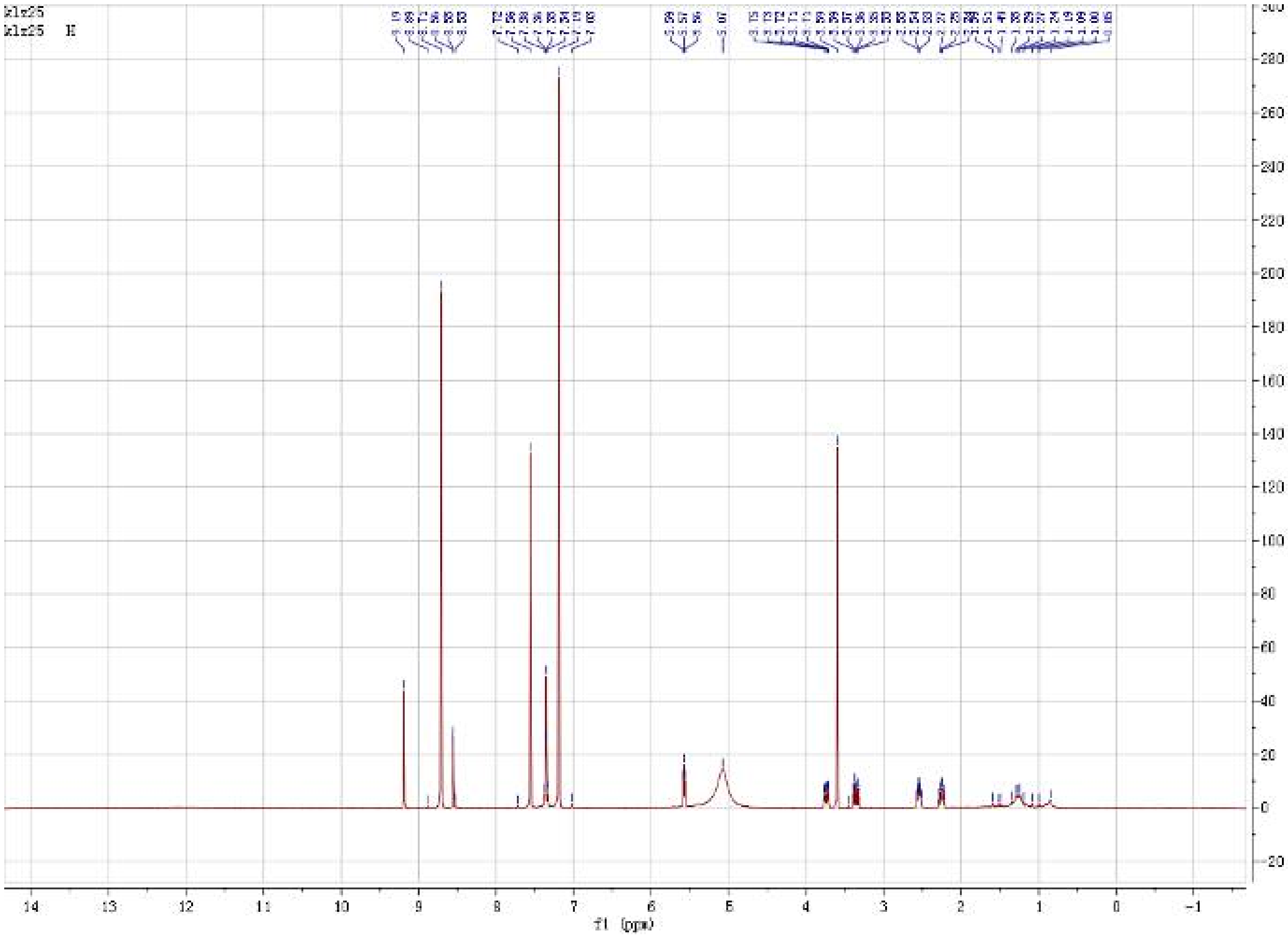

S12. ^{13}C NMR (125 MHz, C_5D_5N) spectra of the new compound sinensine D.

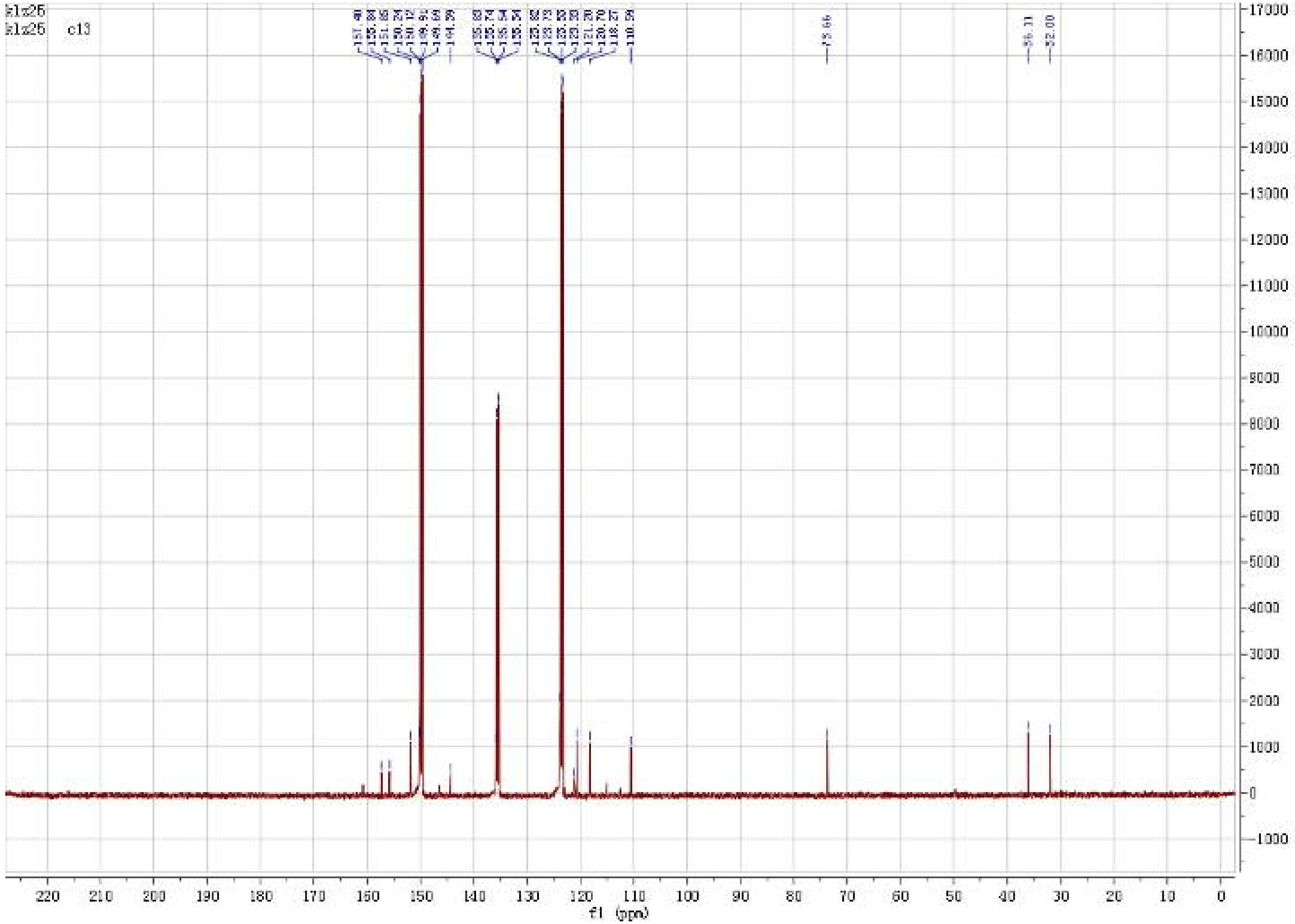

S13. The HMBC spectra of the new compound sinensine D.

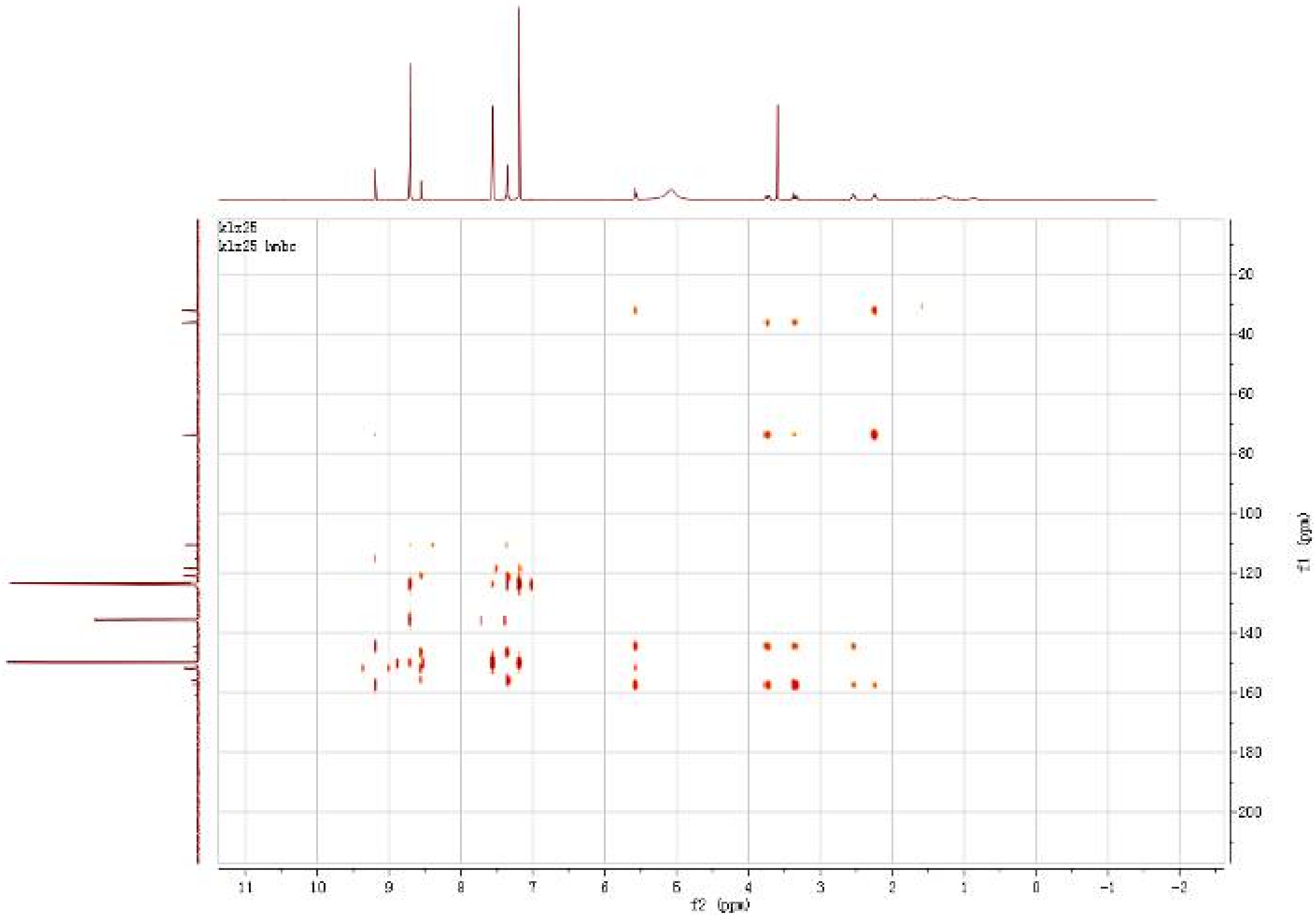

S14. The HSQC spectra of the new compound sinensine D.

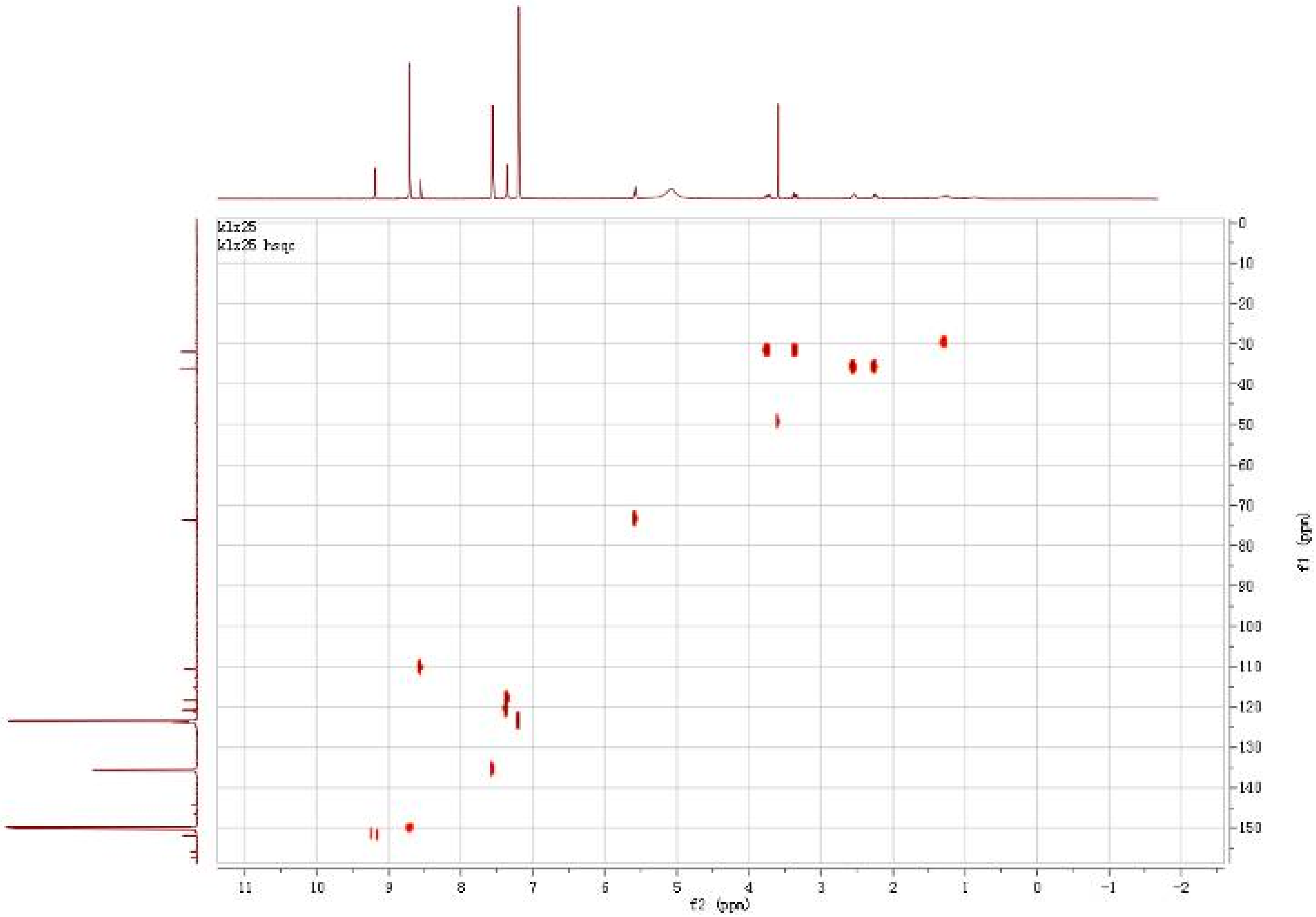

S15. ^{1}H-^{1}H COSY spectra of the new compound sinensine D.

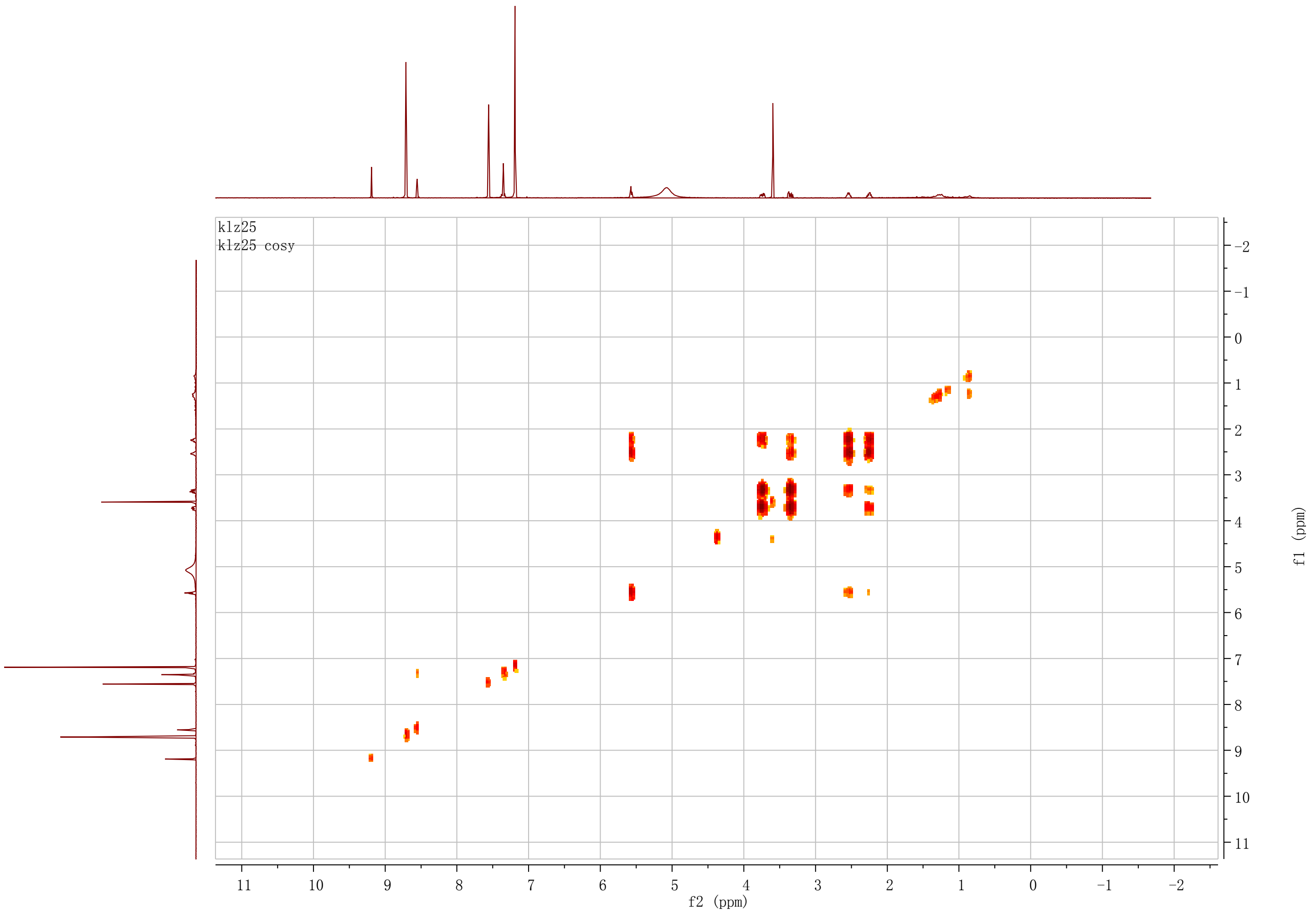

S16. ^{1}H NMR (500 MHz, C_5D_5N) spectra of the new compound sinensine E.

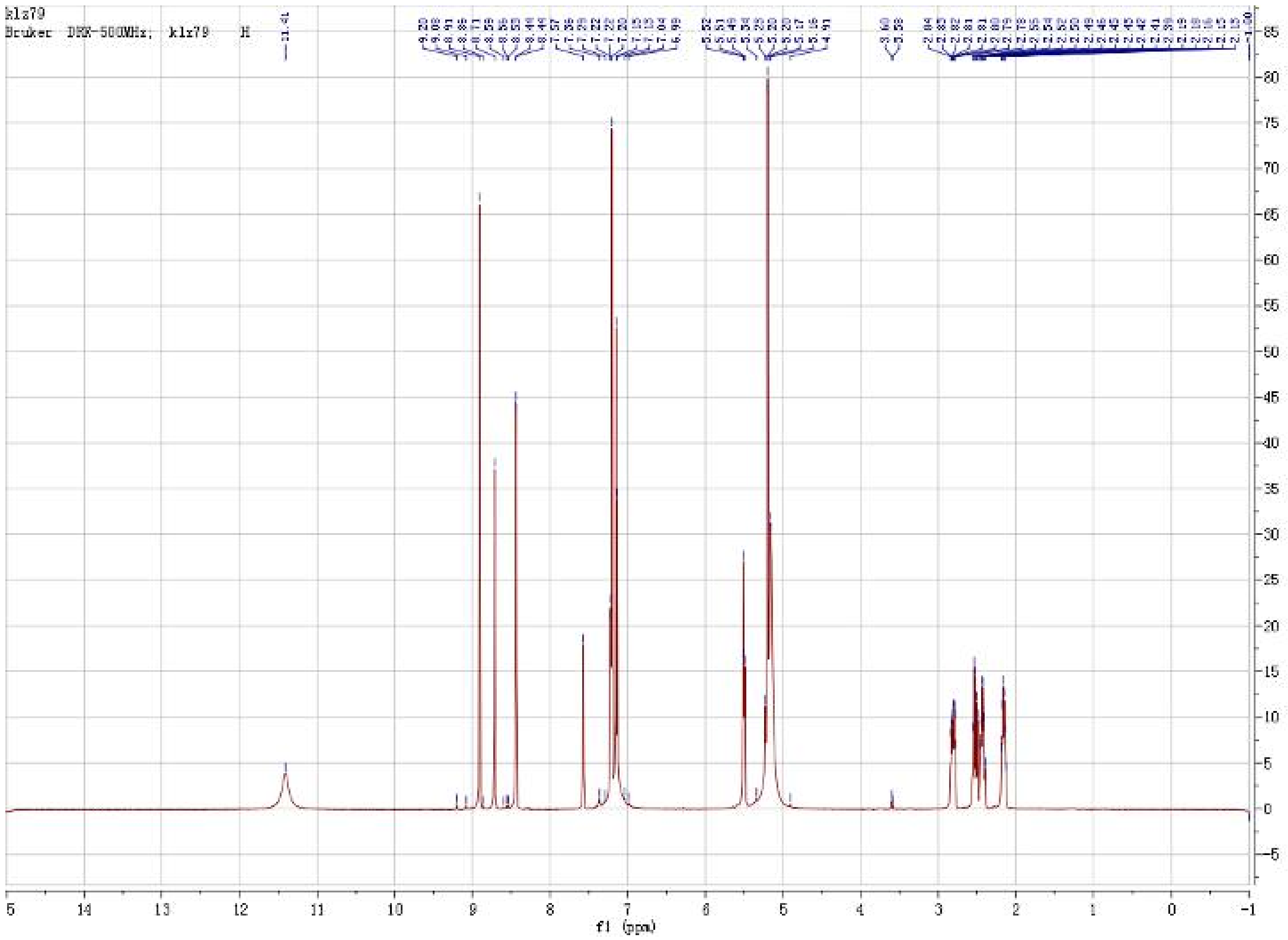

S17. ^{13}C NMR (125 MHz, C_5D_5N) spectra of the new compound sinensine E.

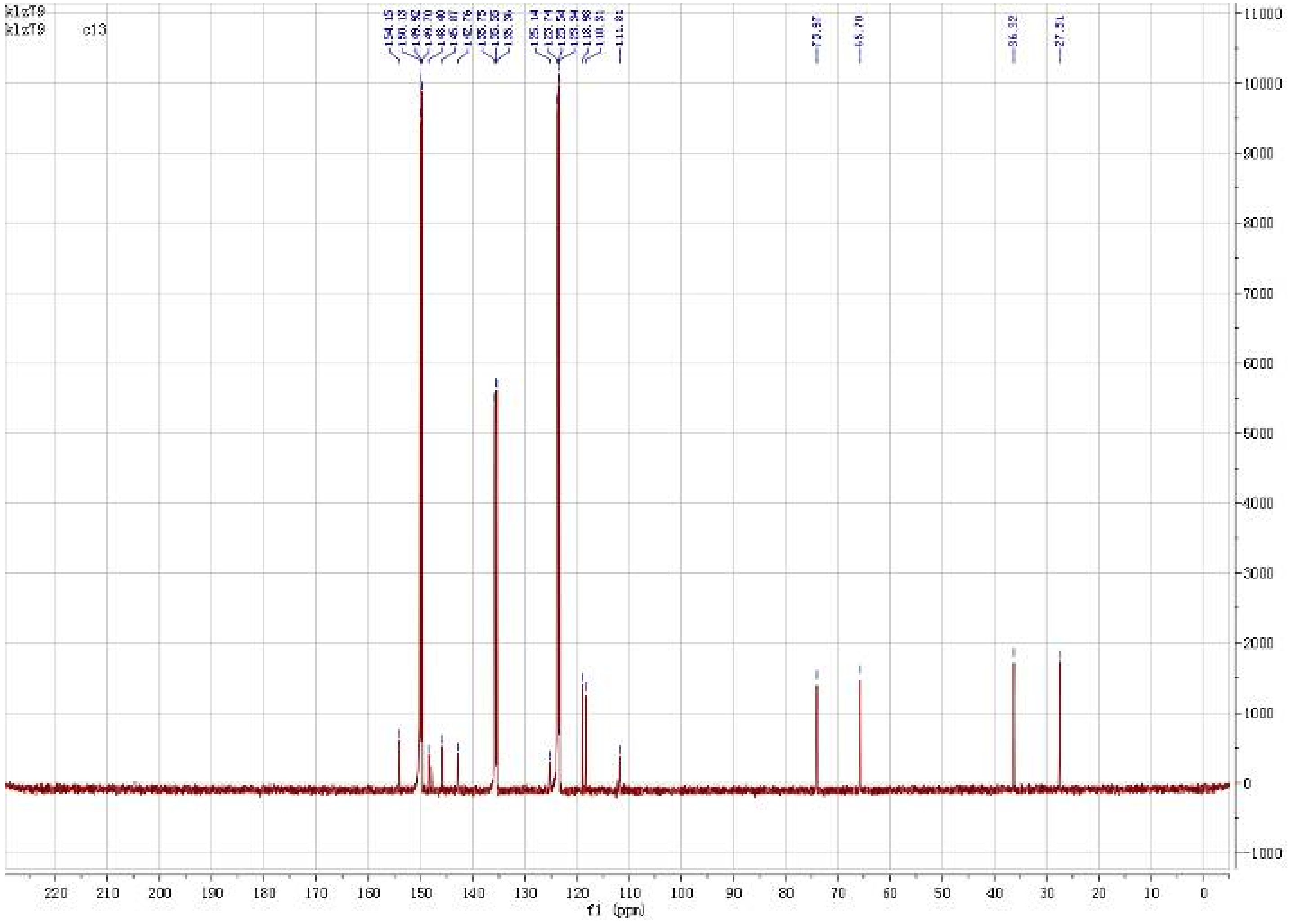

S18. The HMBC spectra of the new compound sinensine E.

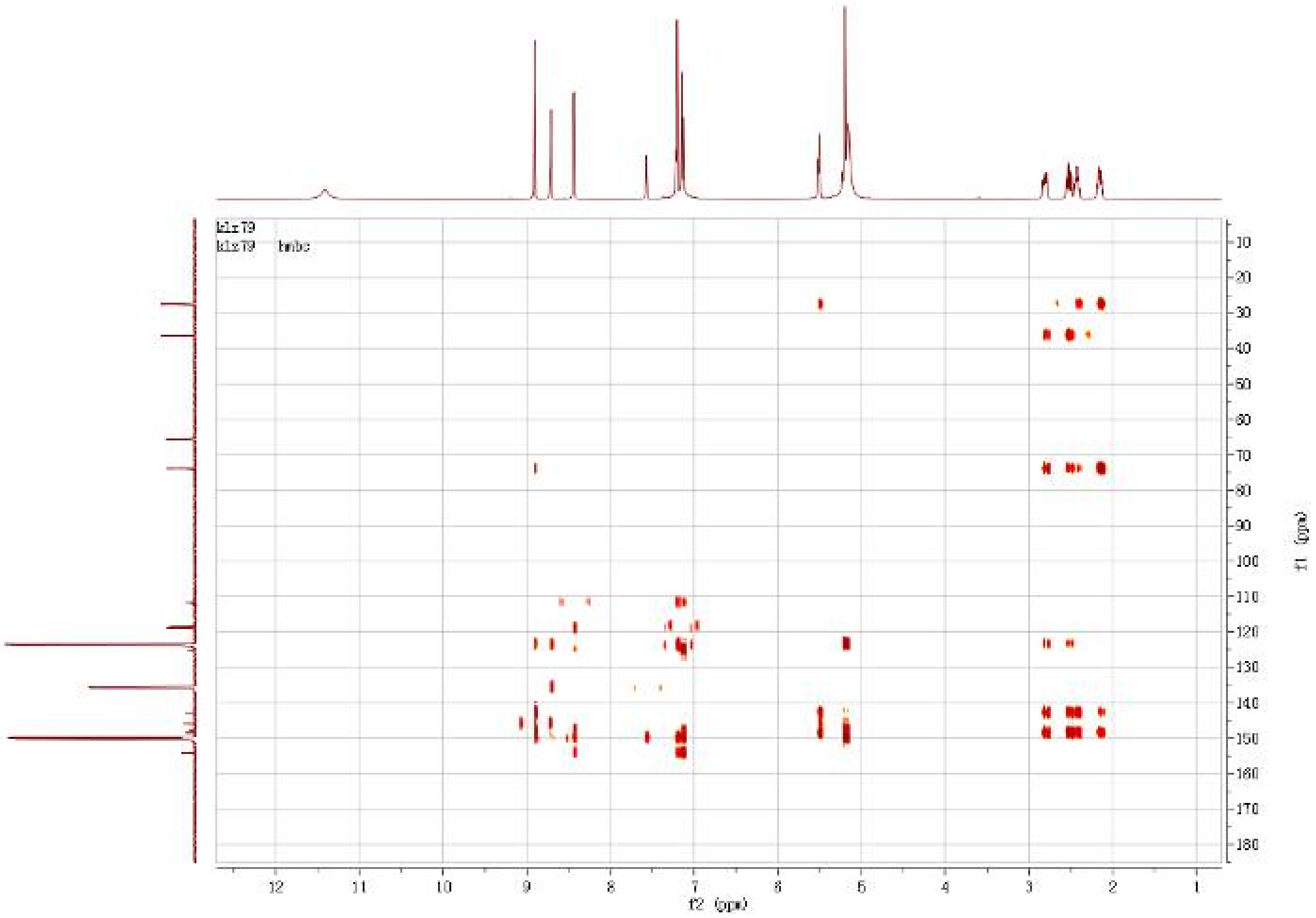

S19. The HSQC spectra of the new compound sinensine E.

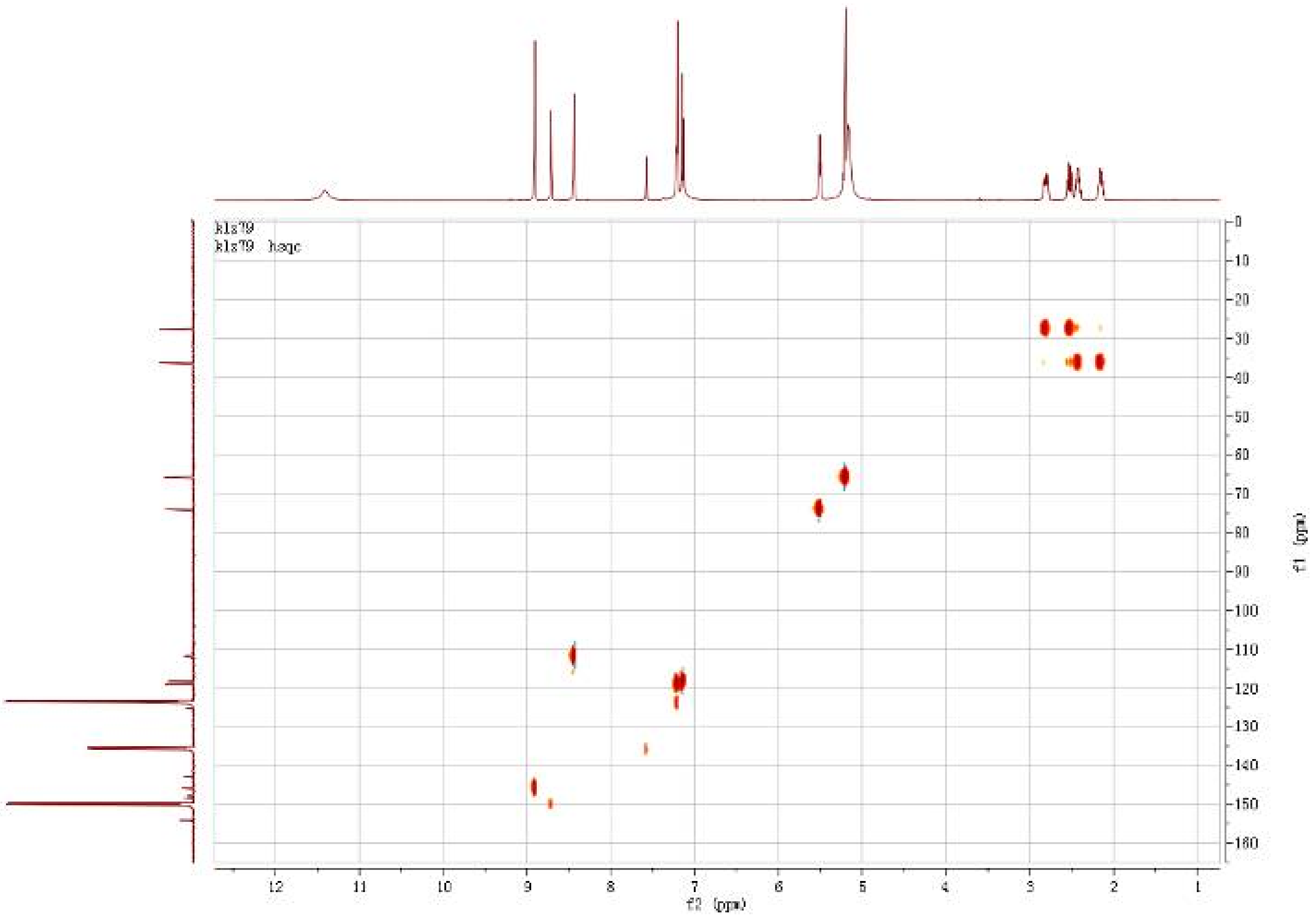

S20. ^{1}H-^{1}H COSY spectra of the new compound sinensine E.

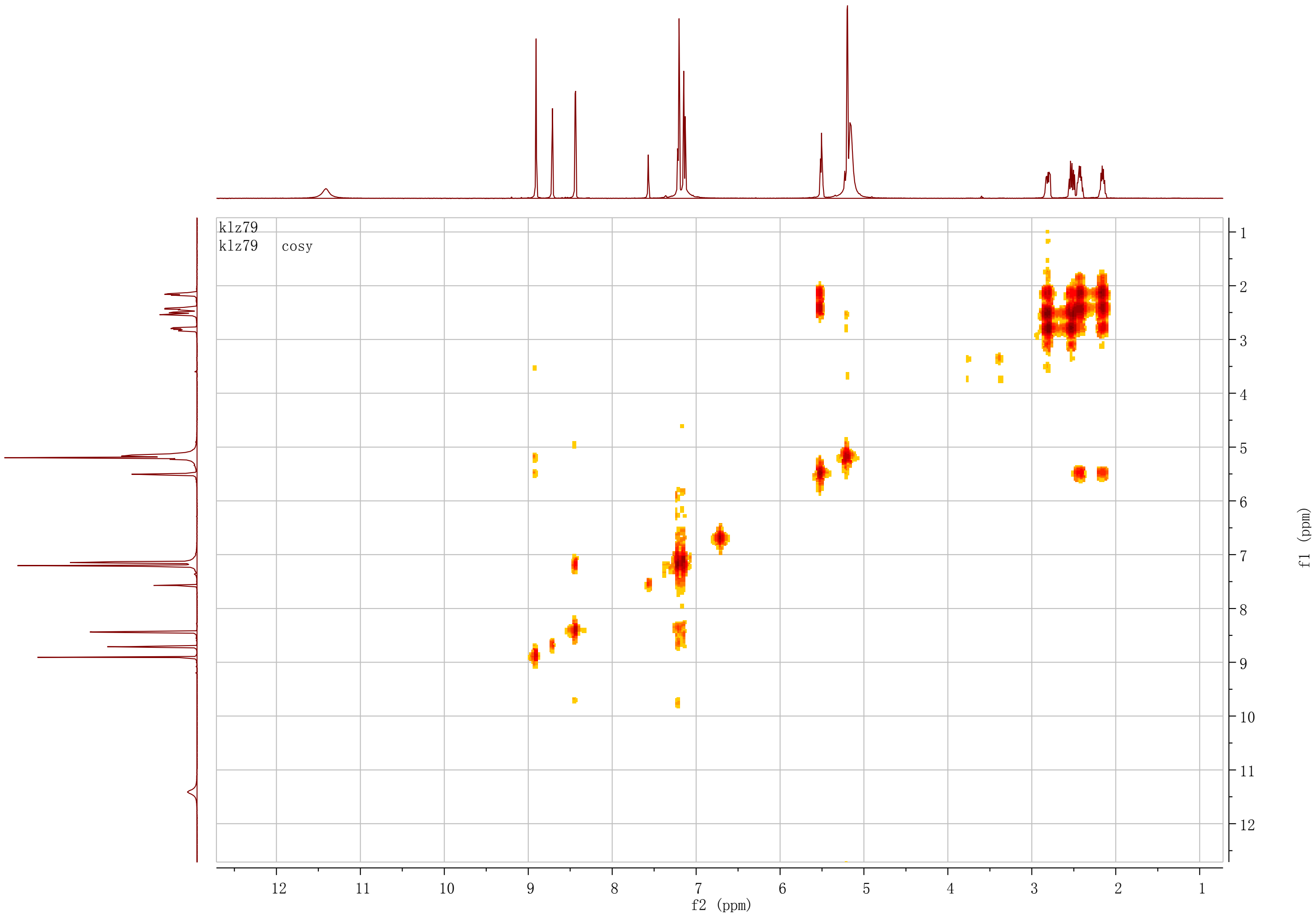

Supplement: Supplementary file 1 — Supplementary material, approximately 708 KB. [file 13659_2011_26_MOESM1_ESM.pdf]
